# Metal oxides and annealed metals as alternatives to metal salts for fixed-ratio metal mixture ecotoxicity tests in soil

**DOI:** 10.1371/journal.pone.0229794

**Published:** 2020-03-05

**Authors:** Mathieu Renaud, Mark Cousins, Kobby Fred Awuah, Olukayode Jegede, Beverley Hale, José Paulo Sousa, Steven Douglas Siciliano

**Affiliations:** 1 Centre for Functional Ecology, Department of Life Sciences, University of Coimbra, Coimbra, Portugal; 2 Department of Soil Science, University of Saskatchewan, Saskatoon, Saskatchewan, Canada; 3 Toxicology Centre, University of Saskatchewan, Saskatoon, Saskatchewan, Canada; 4 School of Environmental Sciences, University of Guelph, Guelph, Ontario, Canada; VIT University, INDIA

## Abstract

In soil metal ecotoxicology research, dosing is usually performed with metal salts, followed by leaching to remove excess salinity. This process also removes some metals, affecting metal mixture ratios as different metals are removed by leaching at different rates. Consequently, alternative dosing methods must be considered for fixed ratio metal mixture research. In this study three different metal mixture dosing methods (nitrate, oxide and annealed metal dosing) were examined for metal concentrations and toxicity. In the nitrate metal dosing method leaching reduced total metal retention and was affected by soil pH and cation exchange capacity (CEC). Acidic soils 3.22 (pH 3.4, CEC 8 meq/100g) and WTRS (pH 4.6, CEC 16 meq/100g) lost more than 75 and 64% of their total metals to leaching respectively while Elora (6.7 pH, CEC 21 meq/100g) and KUBC (pH 5.6, CEC 28 meq/100g) with higher pH and CEC only lost 13.6% and 12.2% total metals respectively. Metal losses were highest for Ni, Zn and Co (46.0%, 63.7% and 48.4% metal loss respectively) whereas Pb and Cu (5.6% and 20.0% metal loss respectively) were mostly retained, affecting mixture ratios. Comparatively, oxide and annealed metal dosing which do not require leaching had higher total metal concentrations, closer to nominal doses and maintained better mixture ratios (percent of nominal concentrations for the oxide metal dosing were Pb = 109.9%, Cu = 84.6%, Ni = 101.9%, Zn = 82.3% and Co = 97.8% and for the annealed metal dosing were Pb = 81.7%, Cu = 80.3%, Ni = 100.5%, Zn = 89.2% and Co = 101.3%). Relative to their total metal concentrations, nitrate metal dosing (lowest metal concentrations) was the most toxic followed by metal oxides dosing while the annealed dosing method was generally non-toxic. Due to the lack of toxicity of the annealed metals and their higher dosing effort, metal oxides, are the most appropriate of the tested dosing methods, for fixed-ratio metal mixtures studies with soil invertebrates.

## Introduction

In soil ecotoxicological research, metal dosing is usually performed using aqueous metal salt solutions in a dilution series [1–6]. This approach allows for a high dosing precision, reduced variability and ease in homogenizing the metals within the soil. Despite these advantages, when dosing with metal salt solutions, salinity may be a cause for concern. Salinity not only affects the chemodynamics of metals in soil increasing their mobility, bioavailability and toxicity [7–10] but can in itself cause toxicity to soil organisms [11,12]. To address these concerns, several authors have proposed and performed the leaching of soils dosed with metal salts to remove the effect of salinity and attempt to increase the realism of laboratory spiked soils compared to contaminated sites [13–15]. A consequence of this soil leaching process is that, in addition to salts, some metal is lost in the leachate. Single metal studies can correct for this by expressing biological response to the realized, rather than nominal, dose. In metal mixture studies, looking at specific ratios of different metal elements in the soil, leaching can be disruptive, because different metals are more or less mobile in soil due to differences in soil-metal-water partitioning [16–18].

In research testing metal mixtures, dosing has been performed with metal salts but without the leaching of dosed soils [1,2,4,5,19,20]. In these studies, only Posthuma et al.[19] acknowledged the importance of salinity by adding sodium chloride to control treatments to balance anion concentrations compared with metal dosed treatments. In mixture experiments where leaching is not performed, and salinity is not corrected, toxicity can be much higher than expected not only due to the increased bioavailability of metals, but also because both metal and salt can contribute to toxicity. Consequently, for fixed ratio metal mixture studies, and considering that leaching can affect mixture ratios, alternative methods for dosing soils must be considered.

This research intends to find a suitable alternative to metal salts when dosing complex metal mixtures. To select an adequate dosing method as an alternative to metal salts it is important to satisfy two criteria, namely the selected dosing method must better retain metal mixture ratios and have an adequate toxicity to soil invertebrates. For this goal, we considered three different methods (metal nitrate salts, metal oxides and annealed metal complexes) for dosing fixed ratio metal mixtures. Each method was evaluated for their total and element-specific metal concentrations as well as their toxicity to standard soil invertebrate test species (*Folsomia candida*, *Oppia nitens* and *Enchytraeus crypticus*) with different sensitivities and routes of exposure, in four different test soils.

Metal nitrate salts were selected to represent the standard practice of metal salt dosing, but which require leaching to remove excess salinity. Metal salts have been used extensively in ecotoxicology with a variety of different metal salts (i.e. metal chlorides [3,19,21,22], sulphates [1,2,6]) including nitrates [6,10,23–25]. Unlike some previous mixture studies in soil, which used a combination of chlorides and nitrates salts for different elements [4,5] we selected the same metal salt (nitrate) for all 5 metals tested in this dosing method. Metal oxides are proposed as an alternative dosing method which does not require leaching and which has also previously been used in soil ecotoxicological research mostly in comparison to metal salt toxicity [26,27]. More recently studies have focused on metal oxide nanomaterials (i.e. [28,29]) but which are considered separately from non-nano oxides used in this study. Annealed metal complexes were selected as a dosing method simulating contamination resulting from a smelting operation and have not been previously tested in the scientific literature except for a similar study on the effects of these dosing methods to soil microbial processes [30]. In terms of their realism and environmental relevance, metal contaminated sites can present a variety of different chemical forms [7], but the annealed metal complexes closely resemble the minerals present at a metal contaminated sites like franklinite and willemite [31,32] whereas oxides and metal salts are less representative but still used in routine in laboratory dosing schemes (especially metal salts) for practical reasons.

To address the adequacy of the different dosing methods in terms of their toxicity, three different soil invertebrate species were considered to cover different routes of exposure. The collembolan *F*. *candida* has been used as a standard test species in soil ecotoxicology for over 50 years and is mostly exposed to contaminants through soil pore-water through the ventral tube and by the ingestion of contaminated pore water and food [33]. Similarly, to *F*. *candida*, *E*. *crypticus* is exposed to contaminants through ingestion but also dermally due to their close contact with soil pore water and lack of protective cuticle [34]. *Oppia nitens* is a relatively new species in soil ecotoxicological research and is exposed to contaminants mostly through ingestion due to their thick sclerotic exoskeleton [35]. However, in juveniles, which lack this exoskeleton, exposure routes can include dermal uptake and affect population performance due to juvenile mortality [6,35]. In terms of sensitivity, *F*. *candida* has a similar sensitivity to *E*. *crypticus* whilst *O*. *nitens* is expected to be less sensitive due to their hard body. Using copper as an example, reproduction EC50 for each species in OECD artificial soil was 477 mg kg^-1^ for *Enchytraeus crypticus* [19], 700 mg kg^-1^ for *Folsomia candida* [24], and 2,896 mg kg^-1^ for *Oppia nitens* [6].

Metal solubility and speciation affect the mobility of metals in soils and consequently their bioavailability and toxicity [7]. In this case metal nitrate salts are expected to have a higher solubility in soil pore water compared to non-soluble oxides or annealed metal complexes [26]. The higher availability in soil pore water (one of the main routes of exposure for invertebrates) implies that nitrate salts should have a higher toxicity than oxides and annealed metal complexes. However, the literature is not always consistent in terms of the relative toxicity of oxides versus salts to soil invertebrates [27,28]. For annealed metal complexes, their toxicity is unknown for soil invertebrates, but this method was designed to incorporate metals in soil directly as a mixture, which is the result of a simulated smelting process, thereby increasing the realism of metal mixture dosing schemes for soil ecotoxicology. When tested using soil enzymes activity (ammonia monooxygenase and acid phosphatases activity), the toxicity of similar dosing methods was both soil and enzyme dependant and did not demonstrate a consistent trend [30].

Soils and their properties can also affect the mobility and availability of metals. The most important soil properties affecting metal bioavailability and toxicity are pH, cation exchange capacity, organic carbon and clay content [7,14,36,37]. Therefore, four different soils covering a range of these different soil properties were selected for method evaluation.

## Methods

All experiments were performed with four different Canadian soils. After collection, all soils were air dried and sieved to <2mm particle size before storage. Soil properties are presented in Table 1. Two of the soils (3.22 and WTRS) were reference soils collected close to mining sites in Flin Flon, Manitoba. Elora soil was collected in Elora, Ontario, while KUBC was a mixed soil from an agricultural research field in Saskatchewan (Kernen) and a soil from Iqaluit, Nunavut (UBC) mixed in a 1:1 ratio. In all cases, private land owners provided permission for soil collection from the sites. Neither endangered or protected ecological species nor humans were sampled for this study.

**Table 1:** Soil properties and closest soil type classification in soil PNEC calculator.

Soil	pH-Cacl	CEC (meq/100g)	Organic C (g/kg)	Clay Content (g/kg)	Water Holding Capacity (ml/g)	Closest soil type in PNEC soil calculator [31]
3.22	3.4	8	17	45	0.3	Acid Sandy Forest
WTRS	4.6	16	25	110	0.35	Acid Sandy Arable
KUBC	5.6	28	12	24	0.48	Loamy
Elora	6.7	21	21	200	0.48	Loamy Alluvial

All four soils (Table 1) were dosed with five different metal mixture ratios (Table 2) at a single mixture dose of 4 toxic units using three different dosing methods: metal nitrate salts, metal oxides and annealed metal complexes. The five mixture ratios were selected based on average metal concentrations for each metal in three contaminated sites in Canada (Flin Flon, Sudbury and Port Colborne), the Canadian soil quality guideline for an agricultural soil use (CSQG) and the estimated PNEC in a Clayey Peaty from the Soil PNEC calculator (Clay Peat).

**Table 2:** Nominal metal mixture ratio compositions in mg kg^-1^ dry weight of soil at a dose of 4 toxic units and *Folsomia candida* EC_50_ used in estimating toxic units.

Mixture	Lead (mg/kg)	Copper (mg/kg)	Nickel (mg/kg)	Zinc (mg/kg)	Cobalt (mg/kg)
Ratio 1—Port Colborne	55.6	380.9	1513	162.6	27.8
Ratio 2—CSQG	536.2	482.6	344.7	1532	306.4
Ratio 3—Flin Flon	202.1	618.6	9.2	2223.3	9.2
Ratio 4—Sudbury	2314.4	160.9	297	1196.4	152.6
Ratio 5—Clay Peat	612.1	662.5	395.7	1199.2	353.6
*F*. *candida* EC50	1600[24,25]	700[24,25]	475[3]	750[24,25]	1480[22]

The mixture dose of four toxic units (TU), was calculated based on *Folsomia candida* literature EC_50_ for each metal element (Table 2). *Folsomia candida* was selected as a standard species to define mixture doses, because literature data is available for all 5 metals. The mixture dose of 4 TU was selected as it was a dose expected to cause toxicity to all invertebrates tested in this study. After each dosing procedure, samples were collected for metal analysis and the remaining soil was used in toxicity assessments. In this study mixture toxicity modeling was not addressed and single metal dosing was not performed, toxicity testing was performed with a single mixture dose (4TU) only to determine the suitability of the dosing methods in terms of effects on soil invertebrates.

### Nitrate metal dosing

Aqueous nitrate solutions of lead (Sigma-Aldrich, Pb(NO_3_)_2_ ACS reagent ≥ 99.0%, #228621), copper (Sigma-Aldrich, Cu(NO_3_)_2_−2.5H_2_O ACS reagent, 98%. #223395), nickel (Sigma-Aldrich, Ni(NO_3_)_2_–6H_2_O, purum p.a. crystallized, ≥97%, #72253), zinc (Sigma-Aldrich, Zn(NO_3_)_2_–6H_2_O, reagent grade, 98%, #228737) and cobalt (Sigma-Aldrich, Co(NO_3_)_2_–6H_2_O, reagent grade 98%, #230375) were pipetted individually to each soil from their respective concentrated stock solutions, to reach the intended mixture ratio. Distilled water was then added to each dosed and control soil to adjust soil water content to 50% water holding capacity and all soils were vigorously mixed. Two weeks after dosing soils, the electrical conductivity of soils was measured, and dosed soils were leached using artificial rainwater [38] one pore-volume at a time until conductivity reached control (non-dosed soil) levels. To account for the loss of fine soil particles from leaching dosed soils, control soils were leached once with one pore-volume of artificial rainwater as well. After leaching, the soils were air dried and lightly macerated to break down aggregates.

### Oxide metal dosing

Commercially available metal oxides of lead (Sigma-Aldrich, PbO, ACS reagent ≥ 99.0% #402982), copper (Sigma-Aldrich, CuO, powder <10 μm, 98% #208841), nickel (Sigma-Aldrich, NiO, 325 mesh, 99% #399523), zinc (Sigma-Aldrich, ZnO, ACS Reagent ≥ 99.0%, #96479) and cobalt (Sigma-Aldrich, Co_3_O_4_ powder <10 μm #221643) were used in soil dosing experiments. When necessary oxides were finely ground to a powder using a mortar and pestle. Once ground, oxides were placed on plastic weigh boats in a sealed glass container with an open beaker of nitric acid. Oxides were left in contact with acid vapours for 48 hours to remove any carbonates and subsequently, air dried in a fume hood for 24 hours. Dried metal oxides were then individually weighed at the appropriate concentration for each metal mixture ratio and added to dry soil. Once all metal oxides were added, soils were thoroughly mixed by stirring and shaking and soil water content was adjusted to 50% water holding capacity.

### Annealed metal dosing

Annealed metal complexes were prepared by precipitating and roasting a metal nitrate mixture solution, to simulate the laboratory equivalent of the ash produced from a smelting operation. In this procedure the same individual aqueous metal nitrate stock solutions used for metal salt dosing were combined to create four mixture stock solutions corresponding to each mixture ratio (Table 2). To each mixture solution an iron nitrate solution was added in a 2:1 molar ratio of iron to the sum of the five metals of interest. The addition of iron was performed to increase the precipitation of the 5 metals of interest (Pb, Cu, Ni, Zn and Co). From this mixture solution an initial 25ml were used in the procedure described below to examine metal precipitation rates in the final ash. This preliminary information was used to correct for unprecipitated metals by adjusting their concentration in the mixture stock solution.

Metals were precipitated by increasing solution pH to 7 ± 0.25 with 14.8M ammonium hydroxide. If the pH rose above 7.25, nitric acid was added to correct for the intended pH value. Once the correct pH was attained the tubes were shaken overnight, after which pH was re-checked and adjusted if needed. The final titrated solutions were centrifuged at 400 g for 30 minutes, after which the supernatant was decanted and resulting precipitates were dried in a fume hood for 12 hours. The resulting pellets were roasted at 600°C for 1 hour in a muffle furnace to decompose the metal nitrate bonds [39–41]. Metal content in ashes was determined to check ratio composition by digesting the samples and analysed through ICP-OES. Sample digestion was performed by stirring 0.05 g of the ash in a heated mixture of HF/HNO_3_/HClO_4_ until dry and the residue was then dissolved in dilute HNO_3_.

Despite initial corrections for unprecipitated metals, in the final annealed material there is always slight deviations from nominal metal ratios. In this case the amount of annealed material applied to each soil was calibrated by using the metal within the ash mixture which best matched the nominal ratio. For example, a target mixture ratio could be 25% lead, 50% copper, and 25% nickel, while the annealed metal complexes were 23% lead, 57% copper, and 20% nickel. In this case the mass balance for dosing each soil would be calculated as per the concentration of lead in the annealed material. After the addition of the metals to soil, these were thoroughly mixed to homogenize and incorporate the metals into the soil and soil water content was adjusted to 50% water holding capacity.

### Toxicity tests

The toxicity of the three different dosing methods for each metal mixture and each soil type were assessed using the reproduction of three different soil invertebrate species. These endpoints were determined using standard protocols for *Enchytraeus crypticus* (ISO 16387 [42]) and *Folsomia candida* (ISO 11267 [43]). *Oppia nitens* tests were conducted adapting the procedures of Princz et al. [35].

Prior to all invertebrate testing, soil water content was adjusted to 50% of their respective water holding capacity (WHC). A description of experimental conditions for each test species is presented in Table 3. In short, after the addition of soil and test organisms to each test unit, these were incubated in the laboratory for four weeks under a photoperiod of 16h: 8h light:dark. For the duration of the incubation period, soil water content was maintained by adding distilled water to match initial test vessel weight and test units were fed with granular yeast (*F*. *candida* and *O*. *nitens*) or rolled oats (*E*. *crypticus*). After 4 weeks of incubation, for *F*. *candida* and *O*. *nitens*, the assays were ended by extracting organisms from each replicate using a heat extractor (previously tested for extraction efficiency (> 90%)) and counted using a binocular microscope. For enchytraeids, organisms from each test vessel were fixed in 70% ethanol and stained with Bengal red (200 to 300μL of 1% Bengal red in ethanol) for 24h. After staining samples were wet sieved using a fine mesh (103 μm) and the organisms were counted using a binocular microscope.

**Table 3:** Procedures adopted in reproduction tests with *Folsomia candida*, *Enchytraeus crypticus* and *Oppia nitens*.

	*F*. *candida*	*E*. *crypticus*	*O*. *nitens*
Guideline considered	ISO 11267	ISO 16387	Princz et al. [24]
Test period (d)	28	28	28
Test containers (mm)	29 x 80	29 x 80	29 x 80
Number of replicates per treatment	5	5	5 control 4 treatments
Number of organisms per replicate	10	10	15
Food source	Dry yeast	Rolled oats	Dry yeast
Days of food supply	0, 14th	0, 7th, 14th, 21th	0, 7th, 14th, 21th
Days of aeration and moisture reestablishment	7th, 14th, 21th	7th, 14th, 21th	7th, 14th, 21th
Soil per test container (g DW)	30	20	30

### Metal analysis

Soil samples were collected from bulk dosed soil for chemical analysis to determine total metal concentrations. Total metal concentrations in soil were determined by reverse aqua regia using trace metal grade nitric and hydrochloric acid (3:1 v/v) and following the procedures described by Topper and Kotuby-Amacher [44] and the EPA [45]. For each treatment soil (1 g) was weighed into 60 ml Teflon digestion vessel and 9ml of nitric acid followed by 3ml of hydrochloric acid were added to each digestion vessel. The digestion vessels were then swirled every 30 minutes until no acid fumes from organic matter digestion were observed and digested in an oven overnight at 105°C. After digestion was completed the resulting solution was filtered using Whatman 42 paper and analysed using ICP-AES for the metals of interest. In addition to soil samples, analysis was also performed for a standard reference material [46], recoveries for the SRM for all elements were on average 73% and always above 66%. Since SRM values recovered were lower than expected measured metal concentrations in dosed soils were corrected for standard reference material recoveries for each metal element respectively. Nominal and measured total metal concentrations for each element, dose method, mixture and soil are presented in supporting information (S1 Table).

### Statistical analysis

All data analyses were performed using R version 3.1.3 [47] with the use of organizational packages Rmisc [48] and PMCMR [49] packages.

**Fig 1:**
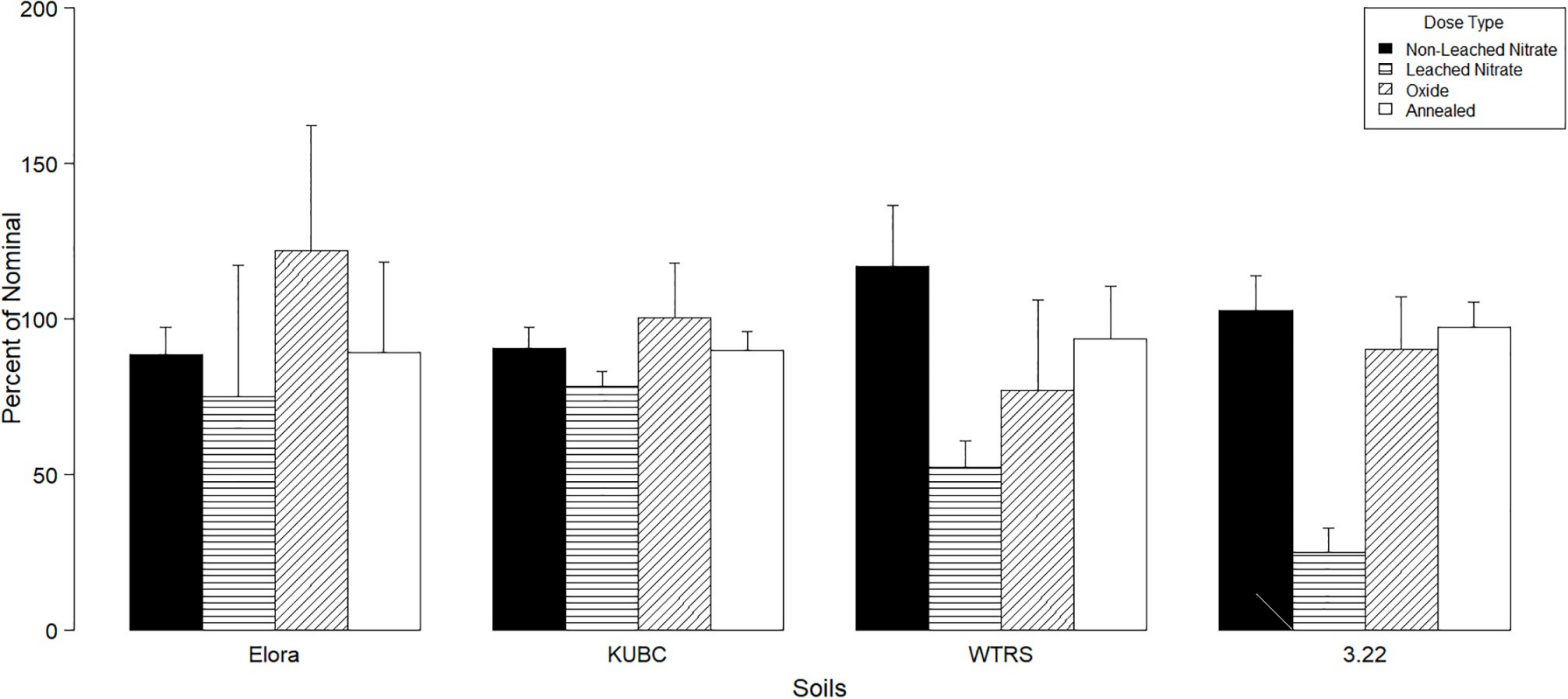
Effect of dosing method on total metal concentrations by soil. Average percentage of nominal total metal concentration (all elements combined) across all mixture ratios (R1—Port Colborne, R2—CSQG, R3—Flin Flon, R4—Sudbury and R5—Clay peat), in each soil according to dosing method. Error bars represent the standard deviation (n = 5).

**Fig 2:**
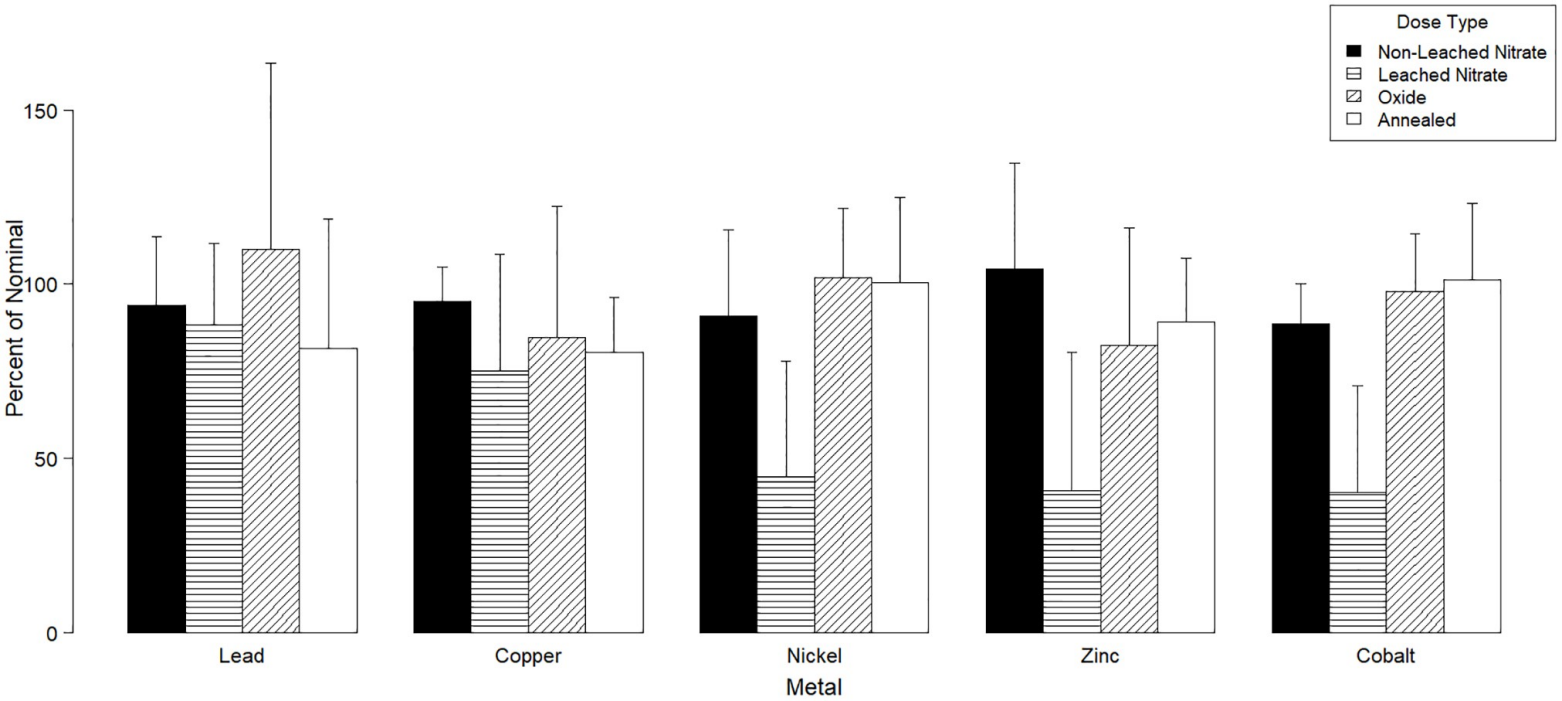
Effect of dosing method on individual metals concentrations. Average percent of nominal concentration combining all soils (Elora, KUBC, WTRS and 3.22) and mixtures (Port Colborne, CSQG, Flin Flon, Sudbury and Clay Peat) of lead, copper, nickel, zinc and cobalt according to dosing method. Bars represent standard deviation (n = 20).

**Fig 3:**
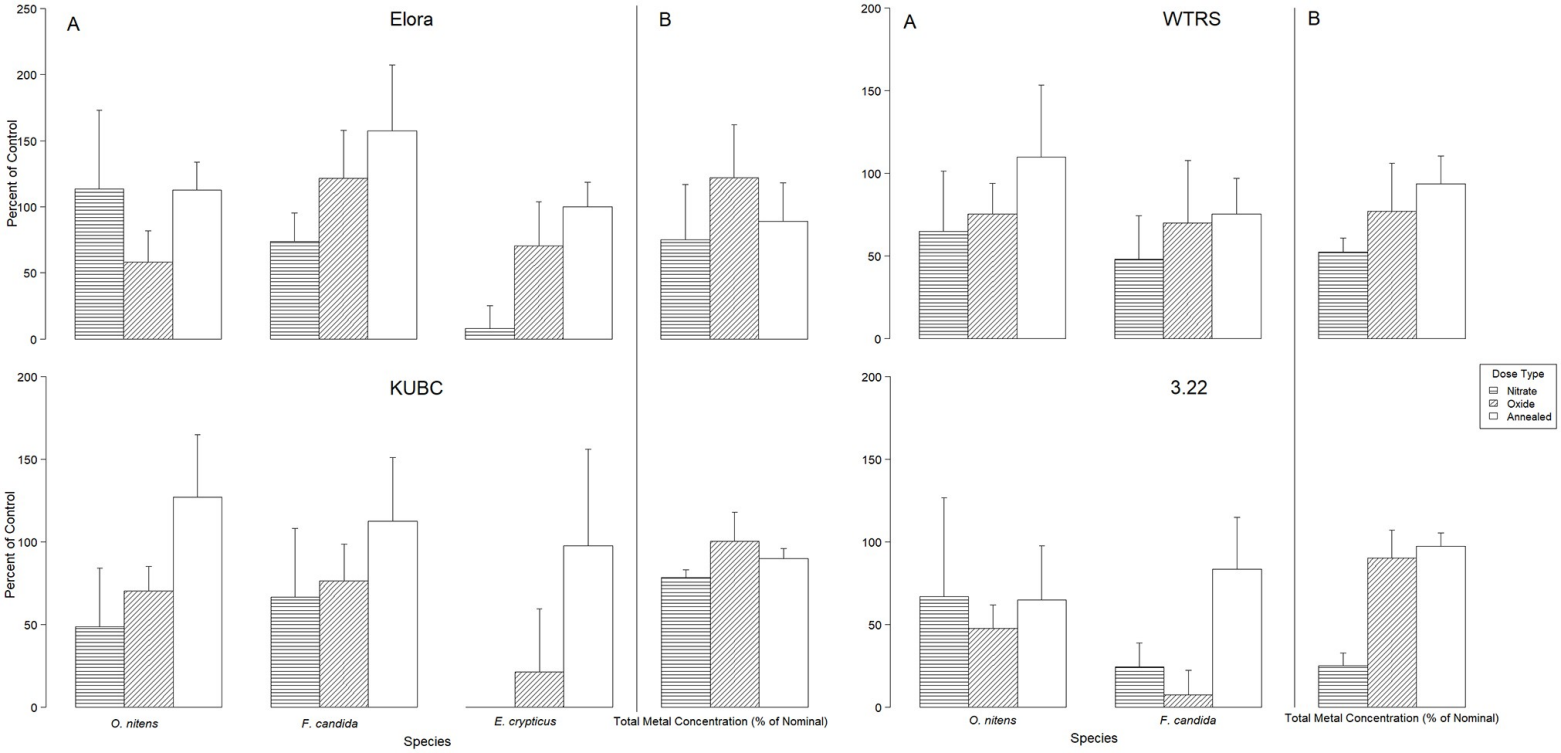
Effect of dosing method on invertebrate reproduction by soil. Average reproduction (as percent of control) for the three species pooled over all metal mixtures for each dosing method and soil (Panel A); total metal concentration as a percentage of nominal dose for each dosing method over all soils and mixtures (Panel B), Error bars represent the standard deviation of the mean (*O*. *nitens* N = 20, *E*. *crypticus* n = 25, *F*. *candida* n = 25 Total metals n = 5).

No statistical analysis is presented in the results (Figs 1–3) because different variables were grouped (soils, elements, mixture ratios) to demonstrate the main effects of the dosing methods on metal concentrations and toxicity. These variables have significant interactions between them, and it would be erroneous to present statistical significances for grouped variables. Results are presented as total metal concentrations (grouping all five elements and mixture ratios) in each soil as a percent of the nominal dose to demonstrate the role of soil properties on total metal concentrations according to dosing method (Fig 1). Individual metals concentrations are also presented as a percent of nominal dose for each element across all soils and mixtures to observe the effects of dosing methods on the concentrations of specific elements affecting mixture ratios (Fig 2). Reproduction results from each species are presented as average percent of control response across all mixtures for each soil and dosing method (Fig 3).

Total metal and individual metal concentration were adjusted for background control soil concentrations and then were normalized to their percentage of target dose (nominal) concentration. This was performed to allow comparisons between soils which have different background concentrations. When adjustments for soil background concentrations resulted in negative values as a result of variation or metal loss from leaching, dosed soil concentration was corrected to zero. In toxicity tests, invertebrate reproduction was normalized to each soil’s average control reproduction.

## Results

Metal concentrations were determined for nitrate metal dosing method before (non-leached nitrate) and after the process of leaching (leached nitrate) to remove salinity, for both oxide and annealed metal dosing methods no leaching was performed because there was no added salt. Soil had a significant effect on the precision or variability of total metal measurements. All soils suffered the same dosing procedures for each dosing method and the same level of effort in mixing metals within the soil. Differences in the magnitude of variability could be the result of differences in soil properties such as soil texture affecting the heterogeneity of metals within the collected soil sample. Elora had the highest variability of the four tested soils followed by WTRS and 3.22 while KUBC was the least variable. Elora and WTRS both had the highest clay content possibly reducing the efficiency of mixing metals within the soil. Excluding non-leached nitrates in the Elora soil, dosing precision was similar between the different dosing methods for each soil. Total metal concentrations for all elements, mixtures and dosing methods are presented in the supporting information (S1 Table).

When comparing non-leached to leached nitrate dosing, acidic soils (WTRS and 3.22) lost more metals through leaching than soils with higher pH values (Elora and KUBC) (Fig 1). In the lower pH Soils, compared to its non-leached counterpart, over 75% (3.22 pH 3.4) and 64% (WTRS pH 4.6) of metals were lost in the leaching process. In contrast, Elora and KUBC with circumneutral pH levels of 6.7 and 5.6 had much lower average percent loss of metals (13.6% and 12.2% respectively) compared to their non-leached treatments. Elora and KUBC also had higher CEC (CEC of 21 and 28 meq/100g respectively) compared to 3.22 and WTRS (CEC of 8 and 16 meq/100g respectively) increasing the binding affinity of metals to soil and reducing metal losses through leaching. For the remaining dosing methods, metal oxide and annealed dosed soils produced similar results to non-leached dosed soils and were not affected by soil except for oxides in Elora where a higher than average recovery was observed. Compared with leached soils, oxide and annealed metal had higher total metal concentrations and were much closer to the target nominal dose in all soils but especially in the more acidic lower CEC soils 3.22 and WTRS.

Leaching selectively removed more than 45% of initially dosed Ni (46.0% lost), Zn (63.7%), and Co (48.4%) (Fig 2). Comparatively, only small losses of lead (5.6%) and copper (20.0%) are observed in the nitrate metal dosing after leaching. In the dosing methods which did not require leaching (oxides and annealed dosing) individual metal concentrations are much more consistent and closer to the target nominal dose, specially for Ni, Zn and Co. For oxide metal dosing, all elements concentrations were higher than leached nitrate dosing, while for annealed lead concentrations were slightly lower. Comparing between oxide and annealed metal dosing, results were similar. Oxides had higher individual concentrations of lead and slightly higher concentrations of copper (Oxide dosing percent of nominal Pb = 109.9%, Cu = 84.6%, Ni = 101.9%, Zn = 82.3% and Co = 97.8%) but annealed metal dosing was slightly more consistent across different elements (Annealed dosing percent of nominal Pb = 81.7%, Cu = 80.3%, Ni = 100.5%, Zn = 89.2% and Co = 101.3%). Overall despite some metals having lower total concentrations towards the target dose, annealed metal dosing had a slightly better consistency for maintaining mixture ratios. Dosing with oxides and annealed complexes produced results similar dosing with metal nitrates prior to leaching the soil (non-leached nitrate metal dosing).

In terms of invertebrate toxicity tests performance, two soils were excluded for *E*. *crypticus*, (WTRS and 3.22) due to insufficient reproduction in controls (<10 individuals). In the two remaining soils (KUBC and Elora), validity criteria were met, average mortality was below 10%, reproduction above 340 juveniles and the coefficient of variation (CV) below 34%. For *F*. *candida* mortality was always below 20% (average 13%), juvenile production above 100 (average 558) in controls. In terms of variation, for the annealed dosing method in the Elora soil the CV in controls was above validity requirements (CV = 64%), however, maintaining or excluding this soil did not change the overall outcome of dosing method toxicity as annealed metals were generally non-toxic. In the remaining controls the coefficient of variation was on average 25% with some soils slightly above the 30% threshold (3.22 annealed– 35%, 3.22 oxides– 31% and KUBC nitrate– 36%). For *Oppia nitens*, no validity requirements currently exist for this species but the coefficient of variation in controls was on average 42%, average juvenile production was 83 and adult mortality was always below 29% per treatment (average 10%).

Soil invertebrate reproduction differed among the three dosing methods relative to their metal concentrations (Fig 3). Relative to their metal concentrations, metal nitrates were the most toxic, followed by metal oxides and annealed metal dosing were the least toxic. Annealed metal dosing was non-toxic at the tested mixture dose in all soils and for all three species except for *O*. *nitens* in 3.22 (average percent control 64%) and *F*. *candida* in WTRS and 3.22 with very small effects (average percent control 75% and 83% respectively). Despite their lower total metal concentration, metal nitrates were always more toxic than oxides to *E*. *crypticus*. *F*. *candida* was also more sensitive to nitrates than oxides, except in the 3.22 soil where metal loss for nitrate dosing was most notable. *O*. *nitens* were more sensitive to nitrates over oxides in WTRS and KUBC but in 3.22 and Elora oxides produced a larger toxic effect. Comparing between species, *E*. *crypticus* was the most sensitive invertebrate species while *O*. *nitens* and *F*. *candida* responses were similar with some exceptions. *O*. *nitens* were more sensitive to oxides and less sensitive to nitrates in the Elora soil compared to *F*. *candida* and *F*. *candida* had a higher sensitivity to both oxides and nitrates in the 3.22 soil. Considering both observed toxicity and metal concentrations, the rank toxicity of the dosing methods from highest to lowest toxicity is nitrate metal dosing, oxide metal dosing and annealed metal dosing. In terms of species sensitivity, *E*. *crypticus* were globally the most sensitive whilst *O*. *nitens* and *F*. *candida* had a similar sensitivity.

## Discussion

Leaching removed metals and the intensity of this loss was affected by soil properties, in particular soil pH and CEC. Acid soils with lower CEC (3.22 and WTRS) lost much more metals than soils with higher pH and CEC values. The role of pH in the mobility of metals has been extensively researched and these results are in accordance with previous literature, where metals, in particular cationic metals have increased mobility in soils with lower pH values [7,15,16]. More recently Dijkstra et al. [50], demonstrated that the pH dependency of metal leaching is in fact a “V-shaped” curve where leaching is lowest at more neutral pH’s (like Elora and KUBC) and highest at both extreme soil pH values. Cation exchange capacity could also contribute to the observed differences between soils. CEC measures the amount of negatively charged sorption sites in a soil, available for cation adsorption [7]. Soils with higher CEC, have a higher capacity to adsorb cationic metals binding them to mineral surfaces leading to lower solubility and bioavailability of metals [7,14,37]. As observed in the mixture results, KUBC and Elora which had a higher CEC, promoted a higher binding of metals to the soil leading to lower metal losses in the leaching process. Soils properties influences the amount of metals lost through leaching but not all metals were equally affected. In this experiment, three elements, Ni, Zn and Co, were lost whereas Pb and Cu were more retained similar to what was previously reported for the mobility of different metal elements in soil [16–18]. Under the current experimental conditions obtaining fixed ratio mixtures is not possible when leaching soils after dosing with nitrate salts.

In this experiment dosed soils were aged for two weeks before leaching, this aging period is short and may have not been long enough to allow metal partitioning within the soil resulting in higher losses during the leaching process. Amorim et al. [51], when testing the role of aging, considered an aging period 60 days following recommendations from a scientific workshop. Using this larger aging period may in fact increase metal retention after leaching, however it would also increase the time required for testing, which can compromise routine testing and be a further disadvantage when compared to oxide and annealed metal dosing methods which do not require extensive aging. Also aging of dosed soils is not commonly considered prior to leaching in soil invertebrate testing even in some research specifically looking at aging and leaching, such as the study of Lock et al. [13], where leaching of Pb(NO_3_)_2_ dosed soils was performed one day after dosing.

Results demonstrated that different elements are lost at different rates as a result of leaching, and the intensity of this loss is dependant on soil properties. Consequently, mixture ratios would change as a function of soil, dose and composition when dosing with the nitrate metal dosing method. While previous research has already demonstrated the differential mobility between metal elements in soils, the goal of this experiment was, not to demonstrate that different elements were lost through leaching at different rates, but rather test if alternative dosing methods would provide an improvement in maintaining fixed metal ratios. In the two alternative methods (oxide and annealed metal dosing) results were more consistent than leached nitrate metal dosing and higher metal concentrations were observed, in particular for Ni, Zn and Co. In both the annealed and the oxide dosing methods there is still some discrepancy towards nominal concentrations (detected values are below 100% of nominal or at times above 100% for individual metals). When dosing soils there is always an error associated with the dosing procedure and the homogenization of metals within the soil but for both oxide and annealed dosing methods this variation towards nominal concentrations is within the range detected for the standard method of nitrate salt dosing without leaching soils.

Comparing between oxide and annealed dosing methods, both methods performed similarly in terms of total metal concentrations excluding the Elora soil where oxides had higher concentrations than expected. In terms of individual metal elements, responses were also similar with the annealed dosing method being slightly more consistent between elements. However, oxide metal dosing was only slightly less consistent than the annealed metal dosing and was still much less variable than leached nitrate metal dosing. Consistency in the oxide dosing method was mostly affected by the higher than average concentration of lead. When dosing with metal oxides, metals were individually added to the soil while for the annealed metal dosing all metals were added together in a single ash product which could explain the better consistency. Considering both oxide and annealed metal dosing produced similar results, dosing with metal oxides seems a more sensible solution for future research due to the substantially higher effort required to create the annealed metal compounds. Future research dosing with metal oxides, should consider combining individual oxides in a mixture prior to their addition to soil to reduce mixture variability across doses. This is similar to preparing a stock solution, but in this case an “oxide mixture stock”, from which all dosing is performed.

In this experiment three different methods were considered: nitrates salts, oxides and annealed materials. Metal nitrate salts were selected to represent a standard dosing procedure with aqueous metal salts. In addition, two other methods were considered which are used in their solid form and which do not contain salts and therefore do not require leaching. Oxides were selected as an insoluble commercially available laboratory grade metal and annealed metal complexes which were created to simulate smelter ash. The recommendation of using metal oxides in fixed ratio metal mixture experiments is based on this selection, however other metal forms of both soluble metal salts (Chlorides) and insoluble metals (Sulphides, sub-sulphides) should be considered for further testing.

### Metal toxicity

Metal nitrate dosing toxicity was similar to oxide and higher than annealed metal dosing despite having the lowest total metal concentrations. In the 3.22 soil, nitrate toxicity was lower than metal oxides, but this is also the soil where the most metals were lost through leaching. The differences in toxicity between dosing methods could be the result of differences in solubility and consequently bioavailability. Compared to metal oxides and annealed metal complexes, nitrate salts were expected to produce higher toxicity. In a similar study, metal salts (after leaching) had similar extractable metal concentrations to metal oxides, in acidic soils, but while oxides only presented extractable Zn concentrations with metal nitrate salts, Zn was the most mobile but a larger proportion of other metals were also mobilized [30]. Annealed metal complexes, the least toxic, also presented mostly Zn and in one case Pb extractable concentrations but only a fraction of the concentrations observed for metal oxides. Also, Awuah et al [30] found that CaCl extractable concentrations for oxide and annealed metal dosing were soil dependant and higher in soils with lower pH, while metal salts had similar extractable concentrations independently of soil. The higher mobilization of metals in lower pH soils does not seem to correspond with toxic response except for the most acidic soil (3.22) where there appears to be a higher toxic response of oxides and annealed complexes compared to the remaining soils, especially for *F*. *candida*. It is also possible that in addition to metal bioavailability soil properties can affect the toxicodynamics of metals, acting as a confounding factor by improving the resilience of organisms to metals in higher habitat quality soils, independently of metal bioavailability as demonstrated for *O*. *nitens* [52].

There has not been previous research on the toxicity of annealed complexes to soil invertebrates, but for metal oxides and metal salts previous research has not been consistent on their relative toxicity. Lock and Janssen [26], using soils dosed with zinc salt, oxide and elemental powders, found a similar chronic toxicity between dosed soils for *Enchytraeus albidus*, *Eisenia fetida* and *Folsomia candida*. Direct comparisons are difficult as mixtures were tested rather than a single metal, but at metal mixture dose of 4 TU, metal oxides and salts (after leaching) had a similar toxicity to *F*. *candida* and *O*. *nitens* with some variations between soils but nitrates were much more toxic than oxides for *E*. *crypticus*. Considering metal loss due to leaching, in this experiment, unlike Lock and Janssen [26], metal nitrate salts are more chronically toxic than oxides. The higher toxicity of metal salts to oxides was also reported in another study, where Zn chloride was more toxic than Zn oxides to *Folsomia candida* [53].

These results demonstrate that metal form and speciation affect metal toxicity, independently of their concentration, potentially due to differences in metal solubility and consequently bioavailability. However, it is also important to highlight that bioavailability is modulated by species traits. In this case, Enchytraeids were much more sensitive to metal nitrate salts compared to both *F*. *candida* and *O*. *nitens*. The higher sensitivity of Enchytraeids is not surprising considering its exposure routes. In addition to oral exposure through pore water, a common exposure route for *F*. *candida*, Enchytraeid’s, can also be exposed dermally due to their soft body and lack of a protective cuticle. This can lead to a higher total exposure and consequently to a larger toxic effect. On the other hand, *O*. *nitens* had a surprisingly similar sensitivity to *F*. *candida* despite their thick exoskeleton. It is possible that *O*. *nitens*, juvenile exposure could lead to mortality and lower reproductive outputs [6,35]. Alternatively, ingestion of soil as part of their feeding behaviour could increase their exposure (which has not been reported for *F*. *candida)* and compensate their more developed external barriers [54]. Further research into the importance of species traits in modulating their exposure to contaminants should be performed specially for more recently standardized test species like *O*. *nitens*.

In all three species, salts which are more soluble in pore water appear to be more toxic than metal oxides, but the correlation between metal solubility and toxicity is not always clear in the literature. Lock and Janssen [26], found that even measuring pore-water concentrations, Zn salts were still more acutely toxic than oxides. Also, Smolders et al. [14] using a large data set of contaminated soils with Cd, Cu, Co, Ni, Zn and Pb found that, pH was a good general predictor of metal solubility in soils, but a poor predictor of toxicity to organisms, and consequently that metal toxicity cannot be inferred from the solubility of metals. In this case and when mixtures are considered, interactions within the soil, the organism and between the different metals are much more complex and further research is required to better understand the role of different exposure routes and metal solubility on their toxicity [55].

Annealed metals were non-toxic to all test species in the tested mixtures at a dose of 4 TU, with only some exceptions where slight toxicity was observed in the more acidic soils (WTRS and 3.22). The choice to create and use this annealed metal mixture was, to an extent, to simulate a smelting operation. This would allow not only the testing of mixtures created as a single compound but also potentially increase the realism of soil dosing for metal ecotoxicology research. Several authors have highlighted that metal salts currently used in ecotoxicology research are not good representatives of contaminated sites and cause a higher toxicity, leading to the recommendation of both leaching and aging soils [13,15]. In this case creating a metal mixture ash as a potential representative of metal forms found in contaminated sites produced a much lower toxicity than expected, considering the known environmental degradation and toxicity of metal contaminated soils. A possible explanation as to why these annealed metal compounds were non-toxic was the use of an iron solution added to promote metal precipitation. The addition of iron increased precipitation by creating bonds between iron and the different metals but might be reducing the availability of the toxic metals. In fact, in a similar experiment, annealed metal complexes only had a fraction of the extractable metals compared to metal oxides and these were restricted to Zn and in one case to Pb [30]. If this is the case, it raises questions as to why toxicity is observed, for instance in smelter sites where minerals like franklinite (iron and zinc mineral—ZnFe^3+^_2_O_4_) are the dominant form of zinc [32]. One hypothesis is that the difference in toxicity is due to weathering. In a contaminated site and particularly in old legacy mining areas, residues from the smelting procedure are weathered down by rain and changing environmental conditions releasing more bioavailable toxic elements into the environment over large periods of time. It could be that these annealed metal mixtures, unlike salts which have a reduction in toxicity due to aging, could have an increase in toxicity over time due to weathering. Further experiments with the annealed dosed soils, looking at toxicity over time should be performed to validate this hypothesis and demonstrate their potential toxicity.

Overall the annealed metal complexes which were expected to be the most realistic approach to simulate metal contaminated sites were generally non-toxic at a mixture dose of 4 TU where effects were expected to occur for all test species. There is a potential for increasing toxicity over time with the weathering of the mineral structure of the annealed metal complexes, but these procedures would not be feasible in routine laboratory experiments. Comparatively metal salts and oxides produced a toxic response to all soil invertebrates, but salts appear to be more toxic than oxides relative to total metal concentrations. Dosing with metal oxides and salts while not as representative of metal contaminated sites are much more practical for routine laboratory dosing regimes. In fact, both metal oxides and salts (in particular) have been previously used in ecotoxicological research and environmental guidelines are currently based on ecotoxicological data with metal salts [56,57].

## Conclusion

Considering that dosing with metal nitrate requires leaching and that this leaching disrupts metal mixture ratios and total concentrations it is not feasible to conduct fixed ratio studies using nitrate salts. The alternative dosing methods (annealed and oxides) which do not require leaching were an improvement in maintaining total and element specific metal concentrations compared to leached metal nitrate dosing and produced similar results to nitrate dosing before leaching is performed. Annealed metal dosing while maintaining more appropriate mixture ratios, did not produce toxic effects within the experimental timeframe of reproduction tests. If our assumptions towards Annealed metals are correct, with the weathering of the annealed complexes an increase in toxicity could be expected over time. In the constraints of standard ecotoxicological tests with fixed ratio metal mixtures, dosing with metal oxides is the most sensible dosing method, retaining fixed mixture ratios and providing adequate levels of toxicity in standard tests. As demonstrated in this study metal speciation is important in determining the toxicity of metals to soil invertebrates as well as their solubility and mobility in soil. As such, in addition to the dosing methods tested in this study other metal forms, using other aqueous salts (i.e. chlorides) or different insoluble metal forms (Sulphides and sub-sulphides) with lower mobility should be considered in further testing.

## Supporting information

S1 Table Nominal and total metal concentrations. Nominal and measured metal concentrations for each dose method, mixture, and soil tested corrected for background metal concentrations. Negative concentrations were adjusted to zero. N/A concentrations indicate contaminated/lost sample not used in analysis.
(DOCX)

S1 File Invertebrate survival and reproduction data. Excel document with invertebrate survival (number of adults) and reproduction (number of juveniles) in each soil, dosing method and mixture ratio, page 1 for *E*. *crypticus*, page 2 for *F*. *candida* and page 3 for *O*. *nitens*.
(XLSX)
