# Supplementary material for: Metal oxides and annealed metals as alternatives to metal salts for fixed-ratio metal mixture ecotoxicity tests in soil
Source: PLoS One. 2020 Mar 5;15(3):e0229794. doi: 10.1371/journal.pone.0229794 (PMC7058289; doi:10.1371/journal.pone.0229794)
Supplement: S1 Table — Nominal and measured metal concentrations for each dose method, mixture, and soil tested corrected for background metal concentrations. Negative concentrations were adjusted to zero. N/A concentrations indicate contaminated/lost sample not used in analysis. (DOCX) [file pone.0229794.s001.docx]

Table 1SM. Nominal and measured metal concentrations for each dose method, mixture, and soil tested corrected for background metal concentrations. Negative concentrations were adjusted to zero. N/A concentrations indicate contaminated/lost sample not used in analysis.

| Dose Type | Soil | Mixture | Lead | Copper | Nickel | Zinc | Cobalt |
| --- | --- | --- | --- | --- | --- | --- | --- |
|  |  |  | (mg kg-1) | (mg kg-1) | (mg kg-1) | (mg kg-1) | (mg kg-1) |
| Nominal Dose | All | CSQG | 536 | 483 | 345 | 1532 | 306 |
| Nominal Dose | All | Flin_Flon | 202 | 619 | 9 | 2223 | 9 |
| Nominal Dose | All | Sudbury | 2314 | 161 | 297 | 1196 | 153 |
| Nominal Dose | All | Port_Colborne | 56 | 381 | 1513 | 163 | 28 |
| Nominal Dose | All | Clay_Peat | 612 | 662 | 396 | 1199 | 354 |
| Nitrate | KUBC | CSQG | 414 | 393 | 286 | 1331 | 236 |
| Nitrate | 3.22 | CSQG | 554 | 437 | 364 | 1505 | 333 |
| Nitrate | WTRS | CSQG | 418 | 396 | 228 | 3197 | 263 |
| Nitrate | Elora | CSQG | 519 | 537 | 339 | 1477 | 274 |
| Leached Nitrate | KUBC | CSQG | 502 | 455 | 261 | 1124 | 200 |
| Leached Nitrate | 3.22 | CSQG | 452 | 167 | 27 | 51 | 13 |
| Leached Nitrate | WTRS | CSQG | 553 | 469 | 158 | 319 | 100 |
| Leached Nitrate | Elora | CSQG | 533 | 497 | 305 | 1399 | 235 |
| Oxide | KUBC | CSQG | 452 | 480 | 326 | 1151 | 318 |
| Oxide | 3.22 | CSQG | 525 | 224 | 312 | 806 | 296 |
| Oxide | WTRS | CSQG | 767 | 331 | 331 | 1213 | 332 |
| Oxide | Elora | CSQG | 930 | 421 | 378 | 1296 | 346 |
| Annealed | KUBC | CSQG | 546 | 397 | 357 | 1354 | 314 |
| Annealed | S3_22 | CSQG | 625 | 428 | 425 | 1534 | 371 |
| Annealed | WTRS | CSQG | 642 | 474 | 441 | 1628 | 384 |
| Annealed | Elora | CSQG | 841 | 602 | 517 | 2047 | 458 |
| Nitrate | KUBC | Flin_Flon | 138 | 549 | 7 | 1959 | 7 |
| Nitrate | 3.22 | Flin_Flon | 178 | 568 | 9 | 1851 | 7 |
| Nitrate | WTRS | Flin_Flon | 209 | 632 | 11 | 3278 | 8 |
| Nitrate | Elora | Flin_Flon | 178 | 580 | 0 | 1986 | 7 |
| Leached Nitrate | KUBC | Flin_Flon | 177 | 553 | 5 | 1482 | 4 |
| Leached Nitrate | 3.22 | Flin_Flon | 202 | 318 | 1 | 166 | 0 |
| Leached Nitrate | WTRS | Flin_Flon | 214 | 563 | 4 | 579 | 4 |
| Leached Nitrate | Elora | Flin_Flon | 202 | 624 | 0 | 1934 | 7 |
| Oxide | KUBC | Flin_Flon | 242 | 648 | 12 | 2055 | 8 |
| Oxide | 3.22 | Flin_Flon | 140 | 430 | 11 | 2793 | 8 |
| Oxide | WTRS | Flin_Flon | N/A | N/A | N/A | N/A | N/A |
| Oxide | Elora | Flin_Flon | 400 | 559 | 12 | 3334 | 10 |
| Annealed | KUBC | Flin_Flon | 166 | 455 | 12 | 1811 | 12 |
| Annealed | S3_22 | Flin_Flon | 198 | 470 | 9 | 1901 | 8 |
| Annealed | WTRS | Flin_Flon | 139 | 384 | 7 | 1520 | 7 |
| Annealed | Elora | Flin_Flon | 171 | 443 | 4 | 1768 | 10 |
| Nitrate | KUBC | Sudbury | 2537 | 152 | 284 | 1033 | 137 |
| Nitrate | 3.22 | Sudbury | 2740 | 118 | 315 | 1180 | 148 |
| Nitrate | WTRS | Sudbury | 2369 | 163 | 287 | 1123 | 145 |
| Nitrate | Elora | Sudbury | 1748 | 145 | 237 | 920 | 108 |
| Leached Nitrate | KUBC | Sudbury | 1897 | 137 | 217 | 805 | 93 |
| Leached Nitrate | 3.22 | Sudbury | 1523 | 22 | 21 | 0 | 5 |
| Leached Nitrate | WTRS | Sudbury | 2247 | 126 | 129 | 164 | 45 |
| Leached Nitrate | Elora | Sudbury | 1 | 0 | 2 | 4 | 1 |
| Oxide | KUBC | Sudbury | 2131 | 121 | 288 | 1175 | 156 |
| Oxide | 3.22 | Sudbury | 2693 | 200 | 285 | 921 | 165 |
| Oxide | WTRS | Sudbury | 1177 | 0 | 129 | 0 | 68 |
| Oxide | Elora | Sudbury | 5343 | 171 | 361 | 1456 | 180 |
| Annealed | KUBC | Sudbury | 2251 | 99 | 274 | 863 | 148 |
| Annealed | S3_22 | Sudbury | 2612 | 108 | 305 | 1005 | 167 |
| Annealed | WTRS | Sudbury | 2509 | 124 | 298 | 1081 | 164 |
| Annealed | Elora | Sudbury | 2143 | 116 | 235 | 962 | 135 |
| Nitrate | KUBC | Port_Colborne | 38 | 405 | 1391 | 153 | 24 |
| Nitrate | 3.22 | Port_Colborne | 57 | 394 | 1720 | 221 | 29 |
| Nitrate | WTRS | Port_Colborne | 84 | 396 | 1615 | 204 | 26 |
| Nitrate | Elora | Port_Colborne | 38 | 334 | 1251 | 147 | 24 |
| Leached Nitrate | KUBC | Port_Colborne | 43 | 343 | 1163 | 124 | 18 |
| Leached Nitrate | 3.22 | Port_Colborne | 58 | 172 | 159 | 0 | 1 |
| Leached Nitrate | WTRS | Port_Colborne | 54 | 363 | 755 | 0 | 9 |
| Leached Nitrate | Elora | Port_Colborne | 53 | 450 | 1480 | 183 | 26 |
| Oxide | KUBC | Port_Colborne | 65 | 702 | 1718 | 176 | 29 |
| Oxide | 3.22 | Port_Colborne | 11 | 214 | 1510 | 66 | 24 |
| Oxide | WTRS | Port_Colborne | 53 | 302 | 1495 | 112 | 24 |
| Oxide | Elora | Port_Colborne | 19 | 241 | 1253 | 142 | 24 |
| Annealed | KUBC | Port_Colborne | 15 | 307 | 1575 | 153 | 31 |
| Annealed | S3_22 | Port_Colborne | 1 | 283 | 1630 | 71 | 30 |
| Annealed | WTRS | Port_Colborne | 27 | 301 | 1637 | 152 | 34 |
| Annealed | Elora | Port_Colborne | 11 | 213 | 987 | 182 | 20 |
| Nitrate | KUBC | Clay_Peat | 564 | 620 | 356 | 1031 | 292 |
| Nitrate | 3.22 | Clay_Peat | 658 | 668 | 440 | 1292 | 380 |
| Nitrate | WTRS | Clay_Peat | 578 | 706 | 405 | 1193 | 362 |
| Nitrate | Elora | Clay_Peat | 545 | 655 | 367 | 1156 | 324 |
| Leached Nitrate | KUBC | Clay_Peat | 631 | 670 | 313 | 888 | 236 |
| Leached Nitrate | 3.22 | Clay_Peat | 511 | 204 | 29 | 0 | 15 |
| Leached Nitrate | WTRS | Clay_Peat | 574 | 560 | 152 | 111 | 98 |
| Leached Nitrate | Elora | Clay_Peat | 604 | 651 | 324 | 1035 | 260 |
| Oxide | KUBC | Clay_Peat | N/A | N/A | N/A | N/A | N/A |
| Oxide | 3.22 | Clay_Peat | 776 | 479 | 399 | 685 | 371 |
| Oxide | WTRS | Clay_Peat | 682 | 545 | 374 | 895 | 362 |
| Oxide | Elora | Clay_Peat | 625 | 748 | 470 | 1060 | 389 |
| Annealed | KUBC | Clay_Peat | 468 | 622 | 403 | 1127 | 287 |
| Annealed | S3_22 | Clay_Peat | 505 | 678 | 452 | 1247 | 321 |
| Annealed | WTRS | Clay_Peat | 454 | 587 | 376 | 1101 | 269 |
| Annealed | Elora | Clay_Peat | 384 | 511 | 309 | 972 | 227 |
